# Supplementary material for: Description of Crinitomyces reliqui gen. nov., sp. nov. and Reassignment of Trichosporiella flavificans and Candida ghanaensis to the Genus Crinitomyces
Source: J Fungi (Basel). 2022 Feb 24;8(3):224. doi: 10.3390/jof8030224 (PMC8953626; doi:10.3390/jof8030224)
Supplement: Supplementary file 1 [file jof-08-00224-s001.zip › jof-1598128-supplementary.pdf]

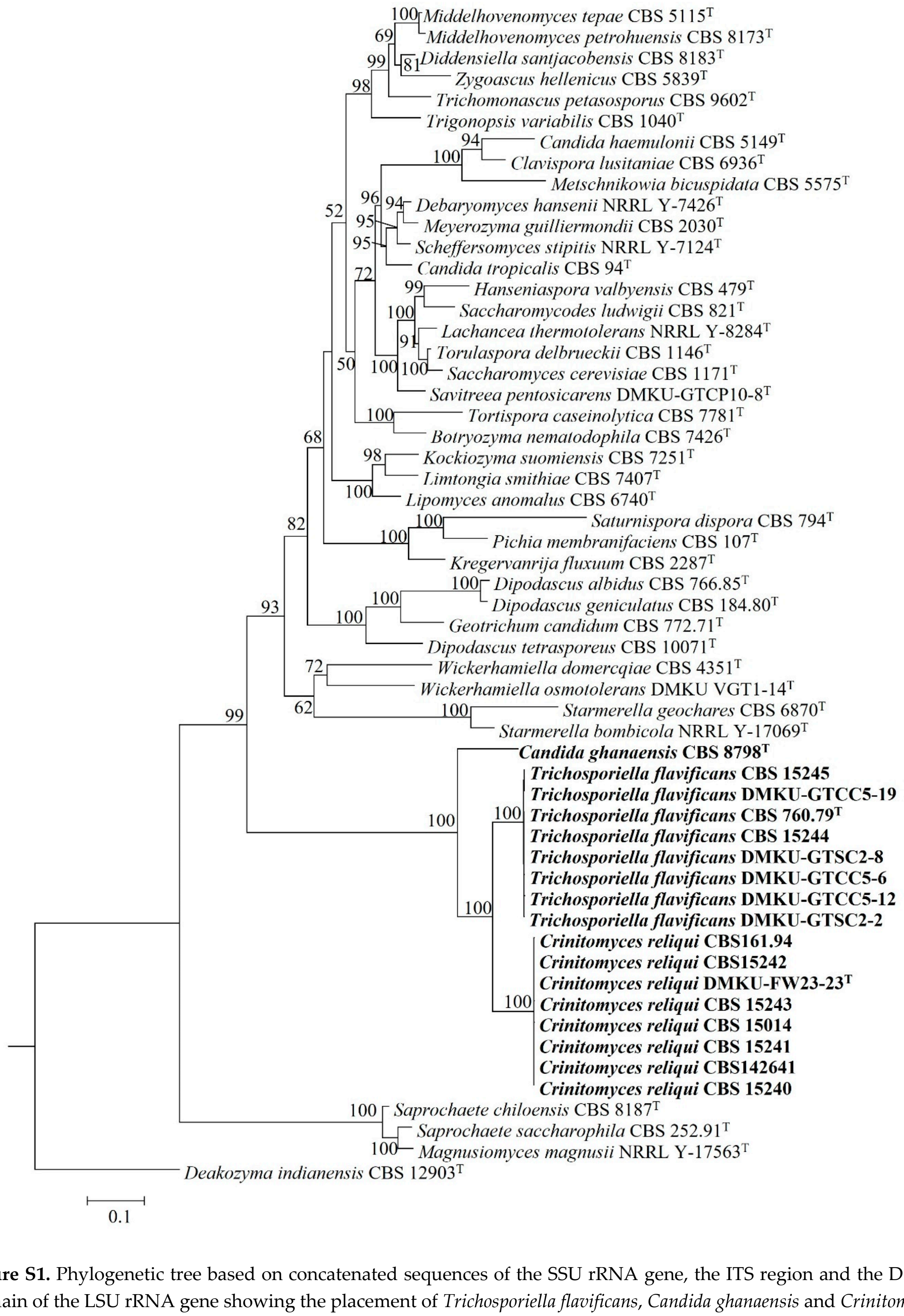

Figure S1. Phylogenetic tree based on concatenated sequences of the SSU rRNA gene, the ITS region and the D1/D2 domain of the LSU rRNA gene showing the placement of *Trichosporiella flavificans*, *Candida ghanaensis* and *Crinitomyces reliqui* within the subphylum Saccharomycotina. The phylogenetic tree was constructed using the maximum likelihood

(ML) method with optimized models for 3 partitions (SSU, ITS region and LSU) in IQ-TREE. The numbers provided on branches are frequencies with which a given branch appeared in 1000 bootstrap replications. Bootstrap values of less than 50% are not shown. *Deakozyma indianensis* CBS 12903^{T} was served as an outgroup species. Bar, 0.1 substitutions per site.
